# Molecular docking analysis of acetylcholinesterase inhibitors for Alzheimer's disease management

**DOI:** 10.6026/97320630019565

**Published:** 2023-05-31

**Authors:** Israa J Hakeem

**Affiliations:** 1 Department of Biochemistry, College of Science, University of Jeddah, Jeddah, Saudi Arabia

**Keywords:** Alzheimer's disease, acetylcholinesterase, bioactive compounds, drug-likeness

## Abstract

Alzheimer's disease (AD) is a neurological disease that is related to aging and is the leading cause of dementia globally. AD has a significant influence on cognitive functions, particularly memory, resulting in a variety of functional deficits. Given the increasing prevalence of AD, there is an urgent need for the development of effective therapeutic therapies. In a quest to uncover a holistic remedy for AD, a total of 41 bioactive compounds derived from three distinct medicinal plant sources were screened to evaluate their potential to inhibit the active sites of acetylcholinesterase (AChE). The insilico screening protocol included 24 licorice-derived compounds, 5 ginkgo biloba-derived compounds, and 11 ginseng-derived compounds. Two compounds (Ginkgolide A and Licorice glycoside D2) were observed to display greater binding energy (BE) relative to the control by interacting with crucial residues in the active site of AChE. Ginkgolide A and Licorice glycoside D2 exhibited BEs of -11.3 and -11.2 kcal/mol, respectively, whereas the control, Donepezil, demonstrated a BE of -10.8 kcal/mol. Further, these compounds exhibit favorable drug-likeness properties. This study suggests that further experimental investigations can be conducted on Ginkgolide A and Licorice glycoside D2 to explore their potential therapeutic applications for AD.

## Background:

Alzheimer's disease (AD) is a prevalent neurodegenerative disorder (ND) that impacts over 46 million individuals worldwide [1-2,3, 4]. It is a fatal ND, and there is currently no known preventive treatment available for it [5,6]. The frequency of AD is expected to rise by three folds in the United States by 2050 [7]. AD was responsible for 121,404 deaths in the United States in 2017, making it the sixth most prevalent cause of death and the fifth most common cause of death among individuals aged 65 or older. Over 16 million Americans, including unpaid caregivers, spent over 18.5 billion hours caring for people with AD or other types of dementia in 2018. The overall costs related to health, long-term care, and hospice facilities for people 65 and older with dementia are expected to reach $290 billion in 2019 [8]. Disruptions in the cholinergic neurotransmitter system are known to play a role in AD-related memory impairment. At present, available treatments aim to target cholinergic synapses in order to enhance synaptic levels of acetylcholine (ACh) and alleviate cognitive deficits related to memory [9]. The cholinergic neurotransmitter system in the brain is critical for cognitive information processing [10], as neurotransmitters are integral components of the neural machinery formed by neurons [11]. Cholinesterase inhibitors (ChEIs) have been approved for the management of symptomatic AD [12]. Tacrine was the initial ChEI authorized by the FDA for the management of AD [13]. However, the medication's usage has been restricted due to its adverse effects, such as gastrointestinal complications and hepatotoxicity [14]. Natural compounds have gained increasing interest as prospective alternatives for synthetic medications due to their perceived safety for human consumption, as they are widely ingested regularly, facilitating clinical approval. A wide range of natural compounds derived from various sources has been proposed for the treatment of ND due to their neuroprotective properties [15]. Licorice, ginkgo biloba, and ginseng have been studied for their potential therapeutic benefits in the treatment of neurodegenerative conditions such as AD, dementia, and Parkinson's disease [16- 17,18]. The goal of this study was to use an insilico approach to evaluate the potential anti-Alzheimer's efficacy of natural compounds derived from licorice, ginkgo biloba, and ginseng targeting the AChE.

## Retrieval and preparation of AChE:

The 3D structure of human AChE in complex with donepezil inhibitor (PDB ID: 4EY7) was retrieved from the PDB [19]. The protein was prepared using Discover Studio and minimized using the universal force field before being used in the virtual screening (VS) procedure. This preparation involved removing water molecules and the co-crystal ligand (donepezil) from the protein.

## Retrieval and preparation of natural compounds:

The natural substances that were utilized in the study were derived from three medicinal plants with an extended record of anti-neurodegeneration research: licorice, ginkgo biloba, and ginseng. These compounds have been retrieved from the PubChem database in .sdf format. These compounds were then minimized using a universal force field (UFF) and saved in pdbqt format for further virtual screening (VS) analysis.

## Structure-Based Virtual Screening:

The prepared natural compound library was screened against the AChE active pocket using the PyRx 0.8 tool [20]. Donepezil was used as a positive control in the VS process. Following the VS process, the highly ranked and fitted compounds in the binding pocket of AChE were further evaluated for 2D and 3D visual inspection. Finally, a comprehensive analysis of interactions between the compound and AChE was performed to select the most stable complex, with an emphasis on lower binding energy (BE) values. The visual inspection of interactions (2D and 3D) was performed using the Discovery Studio visualizer and Pymol.

## Physicochemical and ADMET properties prediction:

Datawarrior tools had been employed to conduct an analysis of all of the compounds that were screened to carry out the preliminary assessment of physicochemical, pharmacokinetic, and drug-like properties [21]. In addition, a web server named ADMETboost was utilized in order to predict the ADMET properties of the best two compounds that were selected [22].

## Result and Discussion:

**Figure 1:**
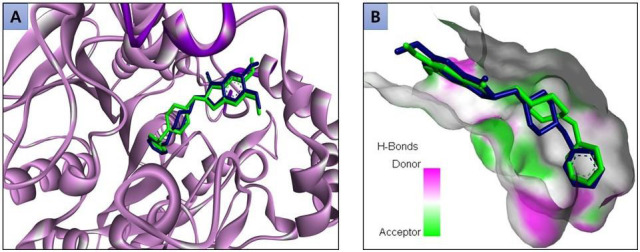
Superimposed binding interaction of donepezil in original PDB structure (green) and re-docked (blue) with AChE protein (A and B).

Ginkgo biloba, ginseng, and licorice have been extensively studied as natural compounds with potential therapeutic benefits for AD and other neurodegenerative conditions. In an effort to discover an all-natural treatment for AD, 41 bioactive compounds derived from three different medicinal plants were tested for their ability to inhibit AChE active sites in a laboratory setting. The screening process made use of 24 compounds that were contributed by licorice, 5 compounds that were contributed by ginkgo biloba, and 11 compounds that were contributed by ginseng. Initially, the docking protocol was validated by redocking the inbound ligand (donepezil) to the active sites of the AChE. The XYZ coordinates were set to -14.01, -43.83, and 27.66, which were obtained from the AChE co-crystal PDB structure. The similarity between the pose of the re-docked complex and the original PDB complex confirms the accuracy of the docking protocol in predicting the binding position of ligands within the AChE protein's binding pocket (Figure 1). In addition to involving donepezil as a positive control, VS analysis of selected 41 compounds revealed several potential compounds with higher or most similar BEs to donepezil (Table 1).

**Figure 2:**
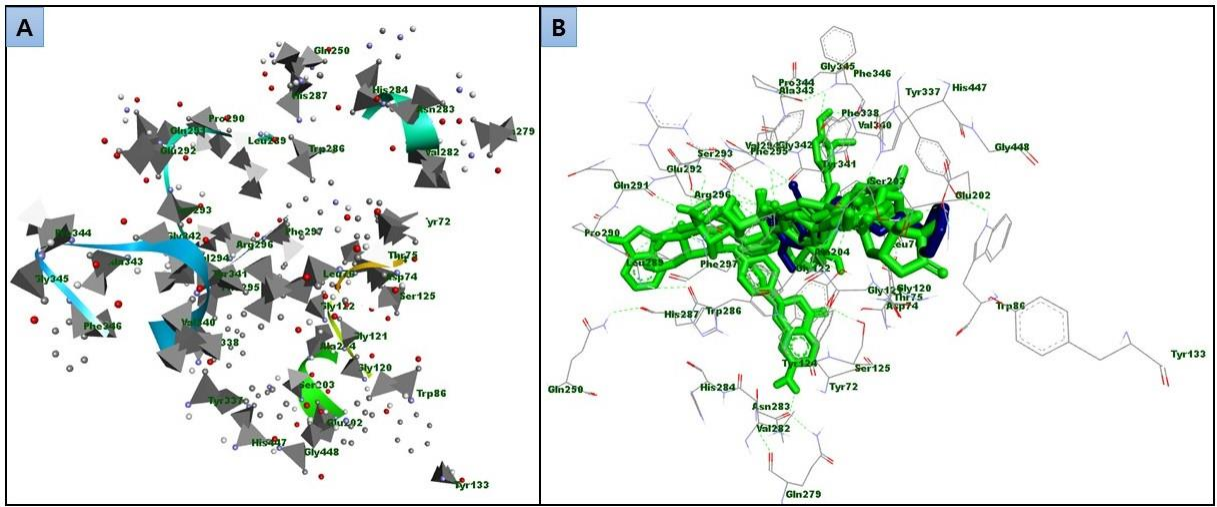
AChE active site residues (A), and the binding of natural compounds as well as the control (donepezil) with AChE (B).

**Figure 3:**
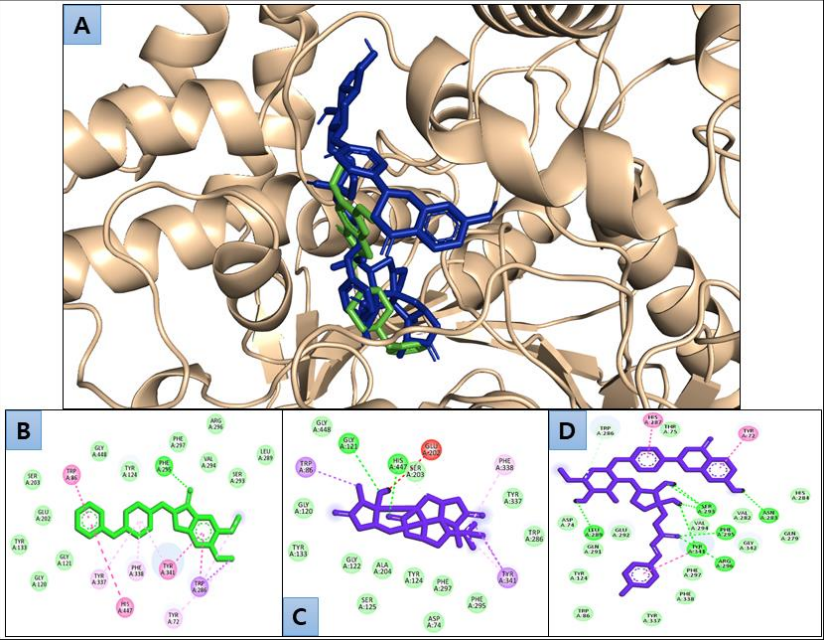
3D visualization of docked poses of Ginkgolide A and Licorice glycoside D2 (blue) and donepezil (green color) (A), and 2D interaction of donepezil (B), Ginkgolide A (C), and Licorice glycoside D2 (D).

During the VS process, centered was on the active site residues that demonstrated a significant amount of interaction with donepezil. Investigations showed that several ligands had interactions with a number of different residues located within the active pocket (as illustrated in (Figure 2). Figure 2A depicts the residues that are present in the active site of the AChE, and Figure 2BFigure 2B illustrates the binding of both natural compounds and the control (donepezil). In this study, two compounds (Ginkgolide A and Licorice glycoside D2) have identified that showed stronger BE to the active pocket of AChE by interacting with its key residues. The findings are based on a comprehensive analysis of binding and visualization of the interactions observed in the docked complexes (Figure 3). Ginkgolide A interacted with Trp86, Gly448, Gly121, His447, Ser203, Glu202, Phe338, Tyr337, Trp286, Tyr341, Phe295, Phe297, Asp74, Tyr124, Ala204, Ser125, Gly122, Tyr133, and Gly120 residues of AChE. Gly121 and His447 residues were H-bonded with Ginkgolide A (Figure 3C). Licorice glycoside D2 interacted with His287, Thr75, Tyr72, His284, Asn283, Gln279, Val282, Phe295, Gly342, Ser293, Val294, Tyr341, Arg296, Phe297, Phe338, Tyr337, Trp86, Tyr124, Gln291, Leu289, Glu292, Asp74, and Trp286 residues of AChE. Leu289, Ser293, Tyr341, Phe295, Asn283, and Arg296 residues were H-bonded with Licorice glycoside D2 (Figure 3D). Phe295 was the common H-bonded residue with Licorice glycoside D2 and the control Donepezil (Figure 3BandFigure 3C, Figure 3D).

The physicochemical properties of all natural compounds were investigated using both Datawarrior tools and Discovery Studio. The values of a variety of physicochemical parameters, such as molecular weight, H-bond donor and acceptor, number of rotatable bonds, aromatic ring, and polar surface area, are presented in Table 2,Table 3, along with their respective predictions.

Further, ADMETboost was used to determine the ADMET properties of the two best compounds (Ginkgolide A, and Licorice glycoside D2). The web server ADMETboost combines a tree-based AI model with a variety of features, such as fingerprints and descriptors. This method made it possible to predict the properties of these compounds accurately. Predictions indicate that both of the identified compounds, which may be referred to as "hits," have the potential to be drug molecules.

## Conclusion:

Numerous compounds have been identified as potential AChE inhibitors, but their FDA approval has been hampered by issues such as poor blood-brain barrier penetration, toxicity, and other drawbacks. This study shows that Ginkgolide A and Licorice glycoside D2 have a high affinity for the active site of AChE and have drug-like properties. However, more research is needed to optimize these compounds as AChE inhibitors, which could potentially provide novel therapies for AD.

## Figures and Tables

**Table 1:** Best ten natural compounds and their binding energy

**PubChem ID (common name)**	**Binding energy (kcal/mol)**
6419993 (Ginkgolide A)	-11.3
42607808 (Licorice glycoside D2)	-11.2
42607807 (Licorice glycoside D1)	-11.1
42607810 (Licorice glycoside C2)	-11.1
42607811 (Licorice glycoside E)	-10.9
6324617 (Ginkgolide B)	-10.9
3152 (Donepezil)	-10.8
54841 (Atomoxetine)	-10.6
101938904 (Licorice glycoside B)	-10.5
73581 (Bilobalide)	-10.4
441921 (Ginsenoside B2)	-10.2
(3152 (Donepezil) is the positive control)

**Table 2:** Physicochemical properties prediction of all the screened compounds

**PubChem ID **	**ALogP**	**MW**	**HD**	**HA**	**RB**	**Num_Rings**	**AR**	**PSA**
54841	3.577	255.355	1	2	6	2	2	0.073
73581	-0.641	326.299	2	8	1	4	0	0.385
3152	4.569	379.492	0	4	6	4	2	0.097
9909368	-0.022	408.399	2	9	1	6	0	0.346
115221	-0.022	408.399	2	9	1	6	0	0.346
6419993	-0.022	408.399	2	9	1	6	0	0.346
6324617	-0.854	424.399	3	10	1	6	0	0.39
10253669	-5.138	459.497	8	9	15	0	0	0.498
42607811	1.614	693.651	7	14	10	7	4	0.357
42607807	1.546	696.651	7	15	11	6	3	0.357
101938904	1.906	696.651	8	15	13	5	3	0.366
42607808	1.546	696.651	7	15	11	6	3	0.357
42607809	1.53	726.677	7	16	12	6	3	0.352
101938907	1.53	726.677	7	16	12	6	3	0.352
101938903	1.89	726.677	8	16	14	5	3	0.361
162343273	1.53	726.677	7	16	12	6	3	0.352
42607810	1.53	726.677	7	16	12	6	3	0.352
21599924	2.014	785.013	9	13	9	6	0	0.262
441922	1.126	801.013	10	14	10	6	0	0.283
441923	1.126	801.013	10	14	10	6	0	0.283
101589043	2.958	806.933	8	15	7	7	0	0.304
452864	2.958	806.933	8	15	7	7	0	0.304
131752455	2.958	806.933	8	15	7	7	0	0.304
129901222	3.208	808.949	8	15	7	7	0	0.303
13457500	3.208	808.949	8	15	7	7	0	0.303
129901221	1.899	820.916	7	16	6	8	0	0.315
86278258	1.899	820.916	7	16	6	8	0	0.315
12889143	2.417	822.932	8	16	7	7	0	0.32
101589724	1.867	822.932	9	16	8	7	0	0.325
14982	2.417	822.932	8	16	7	7	0	0.32
14891570	2.117	824.948	9	16	8	7	0	0.324
14891565	1.327	838.931	9	17	8	7	0	0.341
163463	2.417	888.341	8	16	7	7	0	0.311
86278342	0.604	896.968	9	19	10	7	0	0.349
441934	-0.11	933.127	12	18	12	7	0	0.31
441921	0.267	947.154	12	18	12	7	0	0.304
11679800	0.548	947.154	12	18	13	7	0	0.304
14187172	0.488	985.073	11	21	10	8	0	0.354
6917976	-0.688	1079.27	14	22	15	8	0	0.326
12855889	-0.688	1079.27	14	22	16	8	0	0.326
9898279	-1.198	1109.29	15	23	16	8	0	0.336
(MW: molecular weight; HD: H bond donor; HA: H bond acceptor; RB: number of rotatable bonds; AR: Aromatic ring; PSA: Polar Surface Area)

**Table 3:** Prediction of ADMET properties of 6419993 (Ginkgolide A) and 42607808 (Licorice glycoside D2).

**Molecule Property**		**Value**		**Unit**
		**6419993 (Ginkgolide A)**	**42607808 (Licorice glycoside D2)**	
Absorption				
Caco-2 Permeability		-5.31	-5.72	log(cm/s)
HIA		63	61.91	%
Pgp Inhibition		32.43	38.68	%
log D7.4		1.65	1.79	log-ratio
Aqeuous Solubility		-4.18	-4.41	log(mol/L)
Oral Bioavailability		43.6	37.64	%
Distribution				
BBB		20.54	12.8	%
PPBR		50.18	44.72	%
VDss		3.47	4.17	L/kg
Metabolism				
	CYP2C9	47.92	53.67	%
	CYP2D6	82.26	104.54	%
Inhibition	CYP3A4	36.95	34.38	%
	CYP2C9	30.49	35.17	%
Substrate	CYP2D6	44.53	53.96	%
	CYP3A4	40.51	35.16	%
Excretion				
Half Life		59.75	133.37	hr
CL-Hepa		41.53	39.2	uL min-1(106 cells)-1
CL-Micro		39.44	47.95	mL min-1 g-1
Toxicity				
hERG Blockers		36.32	44.62	%
Ames		43.89	45.98	%
DILI		41.97	50.02	%
LD50		2.16	2.32	-log(mol/kg)
